# Beneficial Impacts of Alpha-Eleostearic Acid from Wild Bitter Melon and Curcumin on Promotion of CDGSH Iron-Sulfur Domain 2: Therapeutic Roles in CNS Injuries and Diseases

**DOI:** 10.3390/ijms22073289

**Published:** 2021-03-24

**Authors:** Woon-Man Kung, Muh-Shi Lin

**Affiliations:** 1 Department of Exercise and Health Promotion, College of Kinesiology and Health, Chinese Culture University, Taipei 11114, Taiwan; nskungwm@yahoo.com.tw; 2 Division of Neurosurgery, Department of Surgery, Kuang Tien General Hospital, Taichung 43303, Taiwan; 3 Department of Biotechnology and Animal Science, College of Bioresources, National Ilan University, Yilan 26047, Taiwan; 4 Department of Biotechnology, College of Medical and Health Care, Hung Kuang University, Taichung 43302, Taiwan; 5 Department of Health Business Administration, College of Medical and Health Care, Hung Kuang University, Taichung 43302, Taiwan

**Keywords:** alpha-eleostearic acid, curcumin, aging, neurodegenerative disease, neurotrauma, CISD2, neuroinflammation, mitochondrial dysfunction, NFκB

## Abstract

Neuroinflammation and abnormal mitochondrial function are related to the cause of aging, neurodegeneration, and neurotrauma. The activation of nuclear factor κB (NF-κB), exaggerating these two pathologies, underlies the pathogenesis for the aforementioned injuries and diseases in the central nervous system (CNS). CDGSH iron-sulfur domain 2 (CISD2) belongs to the human NEET protein family with the [2Fe-2S] cluster. CISD2 has been verified as an NFκB antagonist through the association with peroxisome proliferator-activated receptor-β (PPAR-β). This protective protein can be attenuated under circumstances of CNS injuries and diseases, thereby causing NFκB activation and exaggerating NFκB-provoked neuroinflammation and abnormal mitochondrial function. Consequently, CISD2-elevating plans of action provide pathways in the management of various disease categories. Various bioactive molecules derived from plants exert protective anti-oxidative and anti-inflammatory effects and serve as natural antioxidants, such as conjugated fatty acids and phenolic compounds. Herein, we have summarized pharmacological characters of the two phytochemicals, namely, alpha-eleostearic acid (α-ESA), an isomer of conjugated linolenic acids derived from wild bitter melon *(Momordica charantia* L. *var. abbreviata Ser.)*, and curcumin, a polyphenol derived from rhizomes of *Curcuma longa* L. In this review, the unique function of the CISD2-elevating effect of α-ESA and curcumin are particularly emphasized, and these natural compounds are expected to serve as a potential therapeutic target for CNS injuries and diseases.

## 1. Preface

Neuroinflammation is critically involved in the pathophysiology of acute injuries and diseases (including aging and neurodegeneration) in the central nervous system (CNS) [1,2,3,4]. Profound inflammatory responses are characterized by the activation of glial cells, which are primary innate immune cells of the CNS. Neuroinflammation can induce mitochondrial dysfunction. The main feature is that these reactive glial cells produce nitric oxide (NO) as well as reactive oxygen species (ROS) [5]. Both mutually influencing pathogeneses, inflammation, mitochondrial dysfunction, and eventually neuronal function of the CNS [6,7].

Nuclear factor kappa-light-chain-enhancer of activated B cells (NFκB) has been shown to involve in inflammation and mitochondrial dysfunction [8,9]. NFκB activation can be inhibited by a distinctive zinc finger in addition to the iron-sulfur protein, CDGSH iron-sulfur domain 2 (CISD2) [10]. The preventive effect of CISD2 in opposition to inflammation, and abnormal mitochondrial function supports its role to act as a therapeutic target for CNS injuries and diseases.

**Figure 1:**
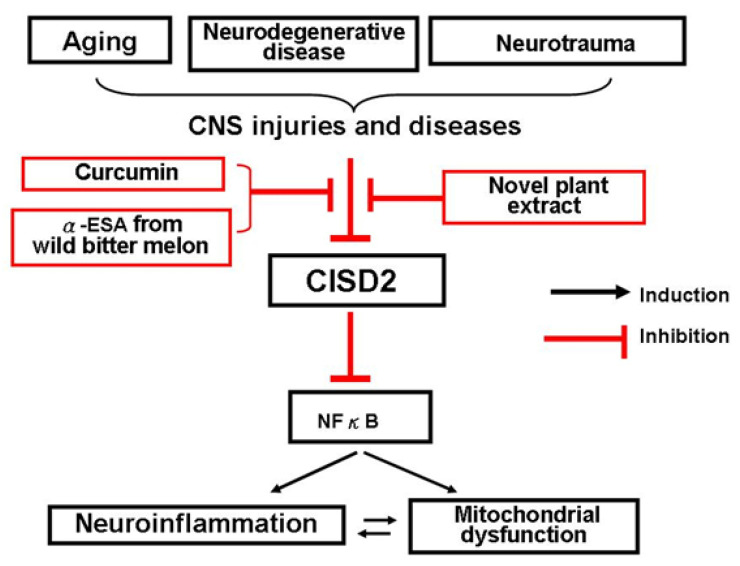
Diagram of CNS pathology–CISD2-NFκB axis. CDGSH iron-sulfur domain 2 (CISD2) expression can be reduced under circumstances of CNS injuries and diseases, such as aging, neurodegenerative disease, and neurotrauma. CISD2 serves as NFκB antagonist. As such, injury-induced decline in CISD2 leads to enhanced NFκB and thereby NFκB-provokes neuroinflammation and mitochondrial dysfunction. CISD2-elevating strategies help to mitigate NFκB-provoked inflammation and mitochondrial dysfunction. Curcumin from *Curcuma longa* L. and α-ESA from *Momordica charantia L. var. abbreviata Ser.* (wild bitter melon) exert anti-inflammatory and CISD2-preservation effects. Any novel plant extracts able to exhibit neuromodulatory effects on this axis of CNS pathology–CISD2-NFκB can be considered to be applied in CNS injuries and diseases.

CISD2 expression level is decreased during CNS injuries and diseases [10,11,12,13]. As a controller of NFκB activation, CISD2 attenuation leads to an exaggerated NFκB activation. This review has focused on the neuromodulatory effects of phytochemicals, such as alpha-eleostearic acid (α-ESA) from *Momordica charantia* L. *var. abbreviata Ser.* (Cucurbitaceae) (commonly named as wild bitter melon, WBM) [10], and curcumin from *Curcuma longa* L. (Zingiberaceae) [12,13], along with the emphasis of CISD2-elevating effects of these natural compounds. Natural medicine beneficial to CNS pathology-NFκB-CISD2 axis (Figure 1) can be recommended to treat CNS injuries and diseases.

## 2. Innate Immune Cells in the CNS—Microglia and Astrocytes

In general, microglia and astrocytes are resident glial cells in the CNS, and these two groups of cells can be activated in response to CNS injuries and diseases, consequently causing local inflammatory response, i.e., neuroinflammation [14]. As with peripheral macrophages, microglia are embryologically derived from myeloid progenitors, whereas astrocytes are derived from neuroepithelial precursors [15].

To maintain CNS homeostasis, microglia and astrocytes provide support and supply nutrition for neurons. Microglia provide neurotrophic support for neurons and mainly mediate immune responses to stabilize the CNS [16]. Astrocyte-mediated neuroprotection has been proposed to be due to the limitation of neuronal death from excitotoxins and oxidants [17], maintenance energy metabolism [18], and the regulation of osmolarity for volume control [19]. Under environmental insults in the CNS, beneficial phenotypes (anti-inflammation) of M2 microglia and A2 astrocytes can be excessively stimulated and potentially switched to detrimental phenotypes (pro-inflammation) (M1 or A1, respectively) [20].

## 3. Diverse Causes of Glial Activation in CNS Injuries and Diseases 

Glial cells can be activated to undergo neuroinflammation as a consequence of various factors in CNS injuries and diseases.

### 3.1. Aging Process

Aging is widely defined as a decline of physiological integrity and gradual impairment of physiological functions over time, and it remains a major risk factor for neurodegenerative diseases [21,22]. Aging in the CNS has been due to cellular senescence and dysfunctional microglia. Microglial dysfunction results in resistance to immune adaptive regulation and the up-regulation of inflammatory genes. Eventually, the proposed senescent [23,24] or dysfunctional glia produce a number of proinflammatory cytokines [25,26].

### 3.2. Neurodegenerative Diseases

Abnormal changes in the conformation of proteins, caused by environmental or genetic factors, lead to an aggregation of intracellular and extracellular proteins, thereby promoting neurodegenerative diseases [27]. These abnormally folded/aggregated proteins implicated in neurodegenerative diseases are commonly known as: hyperphosphorylated tau, Aβ-peptide (for AD), α-synuclein (for PD), huntingtin (for Huntington disease), and prion (for prion diseases) [28]. 

### 3.3. Traumatic CNS Injuries

The brain and spinal cord can be damaged due to mechanical injuries, i.e., traumatic brain injury (TBI) and spinal cord injury (SCI). Primary trauma leads to structural destruction of vital organs, including cell membrane disruption, myelin and axon destruction, as well as neurovascular injury, which further triggers secondary injuries [29]. Specifically, the activation of astrocytes and microglia in the neuroinflammation process involves a complex mechanism of secondary damage to the CNS [30,31]. In a mouse model of TBI, damage-triggered changes in astrocyte morphology have been shown to be accompanied by alternative localization and aggregation of γ-synuclein, indicating potential neurodegenerative changes following CNS injuries [32].

## 4. NFκB-Driven Neuroinflammation and Mitochondrial Dysfunction Implicated in CNS Injuries and Diseases

Under environmental stimuli in CNS, the aggregation of pattern recognition receptors (PRRs), including damage-associated molecular patterns (DAMPs) or pathogen-associated molecular patterns, activates M1 phase microglia and A1 phase astrocytes [33], which in turn enhance neuroinflammation [34]. Pathological mechanisms of glia-mediated neuroinflammation induce the production of several neurotoxic proinflammatory mediators, nitrogen species, and reactive oxygen species (ROS) [35].

Mitochondrial functions such as mitochondrial dynamics, mitochondrial membrane permeabilization, and oxidative phosphorylation can be impaired under the circumstances of neuroinflammation [36,37]. These mitochondrial-derived DAMPs, mitochondrial debris, mitochondrial DNA, and cardiolipin trigger the excitation of NACHT, LRR, PYD domains-containing protein 3 (NLRP3) inflammasomes [38], pro-caspase-1 activation, and production of proinflammatory mediators [39]. This so-called vicious cascade induces the release of even more mitochondrial DAMPs, enhancing further inflammasome activation and more extensive glial neuroinflammation [40].

As a critical transcription factor, NFκB can modulate the transcription of genes in innate and adaptive immunity, proinflammation, cell proliferation, and cellular apoptosis [41]. Neuroinflammation [42,43] and mitochondrial dysfunction [44] are both essential main pathogeneses involved in CNS injuries and diseases, and are provoked by NFκB. Specifically, detrimental insults in the CNS can turn on the IκB kinase (IKK) complex, causing phosphorylation and degradation of inhibitor of κB (IκB). IκB degradation leads to the unmasking of the nuclear localization signal (NLS) in p65/p50 or p50/c-Rel dimers, nuclear translocation of NFκB from cytoplasm, and proinflammation [41]. Furthermore, in mitochondria, NFκB has been shown to regulate mitochondrial function [9], including mitochondrial dynamics, activities of respiratory complexes for electron transport chain, and apoptosis [9]. NFκB has been indicated to regulate the activities of NLRP3 inflammasomes [45]. 

To summarize, neuroinflammation and mitochondrial dysfunction underlie the pathological mechanisms of CNS injuries and diseases. Hence, treatment strategies against the two NFκB-provoked pathologies could be potentially beneficial.

## 5. CISD2 as a Promising NFκB Antagonist

Our research team has demonstrated that CISD2 can block the stimulation of NFκB. Augmented DNA binding activity of the NFκB p65 subunit and nuclear translocation of NFκB p65 were verified in siCISD2-transfected EOC microglia (microglial cell lines) [46]. CISD2 slows down NFκB signaling by acting upstream of peroxisome proliferator-activated receptor (PPAR)-β [10]. PPAR-β has been shown to prevent IκB degradation and subsequent NFκB activation [47,48].

As an NFκB antagonist, the protective effects of CISD2 have been illustrated to exert anti-inflammatory and protective effects against abnormal mitochondrial function. 

### 5.1. Anti-Inflammation

#### 5.1.1. In Vitro Neural Cell Model of Aging

Astrocytes from long-term cultures (35 days in vitro, *DIV*) presented a decrease in CISD2 expression along with higher expression of iNOS and normal T cells expressed and secreted (RANTES), when compared to the 7 *DIV* cells [13]. 

#### 5.1.2. In Vitro CISD2 Knockdown Model

Proinflammatory responses are demonstrated by the expressive levels of iNOS and RANTES in siCISD2-transfected neuron-like cells, SH-SY5Y [12,13]. Augmented inflammatory reactions are revealed by an increased M1 microglia polarization (increased expression of transforming necrosis factor-α, IL-1β, iNOS, and COX2), and decreased M2 microglia phenotype (decreased expression of Arg-1, Ym1, and IL-10) in siCISD2-transfected EOC microglial cells [46].

### 5.2. Protection against Mitochondrial Dysfunction

#### In Vitro CISD2 Knockdown Model

Profound mitochondrial dysfunction is detected in the CISD2 knockdown model using siCISD2 in SH-SY5Y neural cells. Attenuated mitochondrial membrane potential DeltaPsi (m), elevated ROS production, enhanced cellular apoptosis, and impaired cell survival are found in this model [13].

As mentioned before, through the inhibition of NFκB, NFκB-driven NLRP3 inflammasomes, and subsequent inflammasome-mediated inflammation, CISD2 can be considered a promising target to treat inflammation and abnormal mitochondrial function.

## 6. NEET Protein Family and Classification

In the family of NEET proteins, this unique amino acid sequence, Asparagine-Glutamate-Glutamate-Threonine, habitually occurs in the carboxyl end of each family member [49,50]. Three- and one-letter codes of the amino acid sequence are Asn-Glu-Glu-Thr and N-E-E-T, respectively. This is how the protein family is referred to.

The specific sequence motif, CDGSH [51], associates with the [2Fe-2S] cluster through coordinates of 3-cysteine (Cys)-1-histidine (His) on the CDGSH domain [52]. The so-called “CDGSH Iron-Sulfur Domain” represents a common feature of the NEET protein family. In humans, this protein family has three members, CISD1-3 [53,54,55]. “CISD” is named by taking the first word of the common feature “CDGSH Iron-Sulfur Domain”.

According to morphological characteristics, the NEET protein family is divided into two categories [56].

### 6.1. Class I NEET Protein

Homodimers with each monomer including one CDGSH domain, e.g., CISD1 and CISD2.

### 6.2. Class II NEET Protein

Monomeric proteins with two CDGSH domains, e.g. CISD3.

The CDGSH motif is highly evolutionarily conserved. It is found in archaea, bacteria, plants, and humans [57].

## 7. Brief Outline of CISD2 and Biology Perse

In humans, *CISD2* is tracked at the long arm of chromosome 4 (q24) [58]. The *CISD2* gene encodes the CISD2 protein. As mentioned earlier, CISD2 acts as the second member of the human NEET family (CISD1-3), and this is how it is named. In the [2Fe-2S] cluster of the CDGSH domain, it mediates the transfer of iron-sulfur clusters or electrons. Thereby, CISD2 serves as a homeostasis regulator under the circumstances of environmental stress.

CISD2 has been shown to regulate pivotal CNS functions such as acid base homeostasis [59] and oxidation state [60]. Cluster transfer leads to the antioxidant effect of NEET proteins [61]. In general, the [2Fe-2S] cluster of NEET proteins is stable structurally in its reduced state [60]. Environmental stress promotes changes in oxidation-reduction status of [Fe-S] clusters, leading to cluster transfer [62,63,64]. The CISD2 protein includes cytosolic, transmembrane, and in-organelle domain [57]. Through the transmembrane helix, each monomer of the homodimeric CISD2 protein can anchor to the outer membrane of mitochondria (OMM) [59]. CISD2 has been found in the other two subcellular locations, such as endoplasmic reticulum (ER) [55] and mitochondria-associated ER membranes (MAMs) [65,66].

CISD2 is known to have protective effects against calcium excitotoxicity, apoptosis, OMM breakdown, and resultant mitochondrial anomaly. Via a combination of CISD2 and Bcl-2 together with the inositol 1,4,5-triphosphate (IP3) receptor, CISD2 showed to suppress excitotoxic Ca^2+^ escalation at the ER [67,68]. CISD2 deficiency has been demonstrated to augment a Ca^2+^ surge in ER and cytoplasm in CISD2 knockout mice when compared with wild-type mice [67]. Moreover, a CISD2-Bcl-2 combination can regulate Bcl-2 to block autophagy or apoptosis in response to stress [69,70]. CISD2 enhances the association of Bcl-2 with Beclin-1, and eventually avoids cellular apoptosis. As vital components of OMMs, CISD2 proteins have been shown to mediate the maintenance of mitochondrial integrity [71]. CISD2 knockout indicates mitochondrial degeneration, autophagy, and subsequent intensification of the aging process in *CISD2*^−/−^ mice. Wolfram syndrome 2 (WFS2), an ER/mitochondria-related disease, was found to be linked to the recessive mutation of *CISD2*. Wolfram syndrome is spotlighted by diabetes insipidus, diabetes mellitus, optic atrophy, and deafness (DIDMOAD). This WFS2 subtype may present clinically as diabetes mellitus, optic atrophy, and bleeding tendency [72].

Interestingly, CISD2 have various synonyms described by scientists as below. Nutrient-deprivation autophagy factor-1 (NAF-1), endoplasmic reticulum intermembrane small protein (Eris), mitoNEET related 1 (Miner 1), WFS2, zinc finger, and CDGSH-type domain 2 (ZCD2) are common terms which can be used interchangeably. These synonyms indicate important functions of CISD2 in terms of its physiology, subcellular locations, and CDGSH motif.

## 8. CISD2 Attenuation in CNS Injuries and Diseases

In situations such as CNS injuries and diseases, the expression level of CISD2 is definitely reduced.

### 8.1. In Vivo Mouse Model of Aging

Compared with the brain and spinal cord of young mice, the expression of CISD2 was found to be reduced in the corresponding organs of aging mice [13]. There is an age-dependent reduction in CISD2 expression in the brain, skin, and skeletal muscle of mice [73].

### 8.2. In Vitro Neural Cell Model of Aging

A long-term primary culture (35 DIV) of astrocytes demonstrated a lower expression of CISD2 and a higher level of pro-inflammatory mediators when compared with the 7 DIV cell group [13].

### 8.3. In Vivo Mouse Model of Acute SCI 

Diminished expression levels of CISD2 were represented in a contused mice spinal cord sustained hemisection [10,12].

### 8.4. In Vitro Lipopolysaccharide (LPS)—Challenged Neural Cells 

Cellular models of lipopolysaccharide (LPS) challenge have been shown to attenuate CISD2 expression in primary astrocytes [12] and ALT astrocytes (astrocytic cell lines) [10], respectively. 

It is worth noting that CISD2 reduction can be demonstrated in a variety of neural pathologies including aging related neurodegenerative diseases and traumatic neurological insults. In the role of an NFκB antagonist, CISD2 attenuation results in an enhanced activation of NFκB, exaggerating NFκB-provoked proinflammation and mitochondrial dysfunction.

## 9. Searching for CISD2-Elevating Strategy from bioactive Phytochemicals: As a Potential Therapeutic Target for CNS Injuries and Diseases

Under the circumstances of CNS injuries and diseases, these insults can abolish CISD2 expression and correspondingly lead to NFκB-provoked inflammation and abnormal mitochondrial function.

Elevation of CISD2 expression augments the inhibitory effects of NFκB activation. NFκB-evoked inflammation and abnormal mitochondrial function underlying the etiology of CNS injuries and diseases are thereby attenuated. Thus, elevation of CISD2 is recommended as a promising therapeutic strategy for CNS injuries and diseases.

Plenty of naturally occurring phytochemicals with protective effects can originate from plant tissues. These bioactive molecules from natural compounds comprise phenolic compounds, e.g., phenolic acids, flavonoids, conjugated linolenic acid (CLNA), nitrogen-containing compounds, carotenoids, lignans, and terpenes. These phytochemicals possess the inhibitory effect of oxidative cascade and thereby serve as natural antioxidants and potential anti-inflammatory agents [74,75]. Below, we address the general characterization of the two categories of bioactive molecules, conjugated fatty acids and polyphenols. With a brief introduction of their protective properties, we extend our discussion to these two phytochemicals, α-ESA (isomer of CLNA) from the seed oil of WBM and curcumin (polyphenolic phytochemical) from the rhizomes of Curcuma longa. The specific CISD2-elevating effect of the two phytochemicals is a newly discovered feature worth being aware of in this review article.

## 10. Polyunsaturated Fatty Acids (PUFA)s

**Figure 2:**
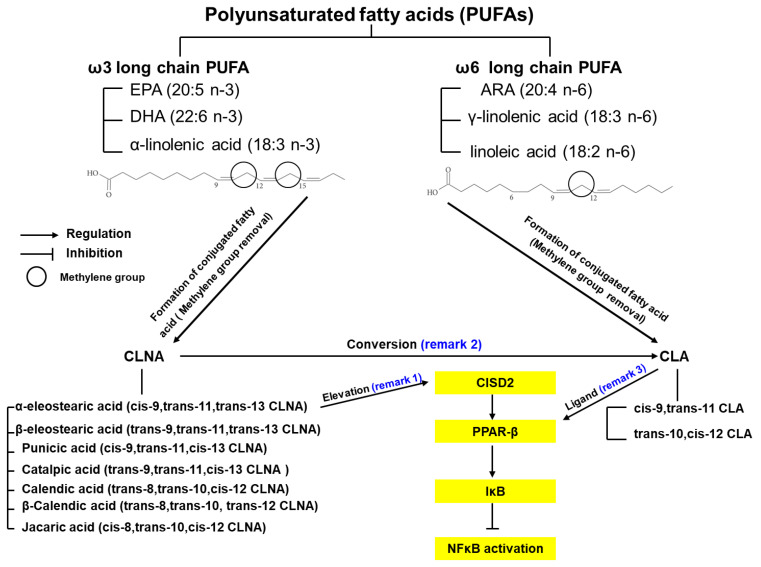
α-ESA, a conjugated linolenic acid (CLNA) isomer, exerts CISD2-elevating effect. Polyunsaturated fatty acids (PUFAs) are mainly divided into two categories, ω-3 and ω-6. Linoleic acid and α-linolenic acid represent the most fatty acids in the ω-6 and ω-3 fatty acids, respectively. By removing the methylene group (represented as a circle) between double bonds in linoleic acid (left sided chemical structure) and α-linolenic acid (right sided chemical structure), conjugated linoleic acid (CLA) and CLNA isomers are derived. As a phytochemical of wild bitter melon (WBM), α-ESA is a CLNA isomer, which can elevate expression levels of CISD2 (indicated as remark 1). CISD2 serves as a PPAR-β regulator at upstream level. α-ESA can be converted to CLA (indicated as remark 2). CLA has been demonstrated to function as PPAR-β ligand (indicated as remark 3). As a result, α-ESA-driven CISD2 activation induces PPAR-β upregulation, causing the inhibition of IκB degradation and NF-κB activation.

Polyunsaturated fatty acids (PUFAs) remain one of the three main constituent elements of fatty acids (saturated, monounsaturated, and polyunsaturated). This category of fatty acids influences the synthesis of cellular membranes and the function of membrane-bound enzymes/receptors; it also provides energy sources for humans [76]. PUFAs include the ω6 and ω3 families, and the main components of each are revealed as follows. Linoleic acid, γ-linolenic acid, and arachidonic acid (ARA) are classified as ω6 PUFAs, whereas α-linolenic acid, eicosapentaenoic acid (EPA), and docosahexaenoic acid (DHA) are cateogorized as ω3 PUFAs [77] (Figure 2). Belonging to the most common PUFA ω6 and ω3 families, linoleic acid and α-linolenic acid (so called essential fatty acids) cannot be manufactured by our own human body, but ought to be recruited from the environment as food intake. Linoleic acid and α-linolenic acid are mainly derived from plants [78]. Linoleic acid can be obtained from most plant seeds in addition to coconut, cocoa, and palm; while on the contrary, α-linolenic acid can be drawn from seeds of flax, rape, chia, perilla, and walnuts as well as through green leafy vegetables.

This ù6 PUFA, linoleic acid, can be transformed into ARA, which potentially enhances proinflammation and increases the risk of cardiovascular disease and metabolic syndrome. On the other hand, ù3 PUFA, α-linolenic acid is metabolized to EPA, which could mediate anti-inflammatory effects [78]. Today, ù6 fatty acids, especially linoleic acids, remain the main sources of PUFAs in the Western diet, accounting for >80% of PUFA intake [79]. Controversy still exists in the role of ù6 PUFAs for their pro- or anti-inflammatory effects. In comparison, beneficial impacts of ù3 PUFAs in obesity and diabetes mellitus have been addressed in detail [80]. Changing eating habits is recommended, such as increasing the ratio of (n-3): (n-6) PUFA in the Western diet, which reduces the occurrence of chronic inflammatory diseases [81].

## 11. Conjugated Fatty Acids

Conjugated fatty acids, as isomers of PUFAs with double bonds in conjugation, have been addressed and have aroused with great interest due to their specific biological activity. This group of conjugated fatty acids, the conjugated linoleic acid (CLA), has been shown to exert superior antioxidant cellular response against oxidative stress when compared to PUFAs [78].

Figure 2 indicates the formation of the two categories of conjugated fatty acids. CLAs and CLNAs are derived from the ù6 and ù3 PUFAs, linoleic acid and α-linolenic acid, respectively. With the removal of methylene group between double bonds in linoleic and α-linolenic acid, CLAs and CLNAs stand for the main dienoic and trienoic derivatives of the PUFAs, respectively [82].

In general, cis-9,trans-11 and trans-10,cis-12 CLA represent the two main categories of CLA isomers [83]. The former isomer represents the main naturally occurring CLA. In contrast, the later isomer shows better tumor-suppressive effects [84]. The following conjugated fatty acids stand for the main categories of CLNA isomers: α-eleostearic acid (α-ESA, cis-9,trans-11,trans-13 CLNA), â-eleostearic acid (trans-9,trans-11,trans-13 CLNA), punicic acid (cis-9,trans-11,cis-13 CLNA), catalpic acid (trans-9,trans-11,cis-13 CLNA), calendic acid (trans-8,trans-10,cis-12 CLNA), â-calendic acid (trans-8,trans-10, trans-12 CLNA), and jacaric acid (cis-8,trans-10,cis-12 CLNA) [85].

Dietary conjugated fatty acids have been shown to benefit human health. Beef tallow (a kind of ruminant fats), milk fat, and dairy products account for the major sources of CLAs [86]. However, the CLA content in these foods is only approximately 1% [87]. CLA can be worked out by alkali isomerization of vegetable oils (such as safflower oil) as dietary additives [88]. In contrast to CLAs, CLNAs are mainly derived from plants and account for more than 70% of plant fatty acids [89]. CLNAs are derived from the triglycerides in the seed oils of the families Cucurbitaceae, Punicaceae, Bignoniaceae, Rosaceae, Chrysobalanaceae, Lythraceae, Balasaminaceae, and Euphorbiaceae [82].

α-ESA and punicic acid are two typical CLNAs found in seed oils. The amount of punicic acid derived from snake gourd (*Trichosanthes anguina*) seed oil account for about 40% [82]. It has been shown that bitter melon oil and tung seed oil contain a large amount of α-ESA, about 60% and 70%, respectively [89]. 

CLNAs have many well-known beneficial effects, covering anti-carcinogenic, anti-adipogenic, anti-inflammatory, and anti-atherosclerotic properties [90]. In rats, α-ESA and punicic acid can attenuate lipid peroxidation and act as an antioxidant [91]. In patients with diabetes, α-ESA has been shown to reduce lipid peroxidation in plasma and erythrocyte membrane [91,92].

## 12. Conversion from CLNA to CLA

The conversion from CLNA to CLA has been addressed. As isomer of linolenic acid, α-ESA, has been shown to metabolize into cis-9,trans-11 CLA in mice [84,93] and rats [87,94]. Punicic acid has been shown to transform to cis-9,trans-11 CLA [95]. The protective effects of CLNAs are related to CLAs, and the pharmacological advantages of CLAs can be considered for CLNAs because of the conversion described above.

## 13. Recommended Dosages of Conjugated Fatty Acids

In the case of a 70 kg human, the suggested daily doses have been addressed as 3–6 g/day [96,97] for CLA and 2–3 g/day for CLNA [96,98]. It is recommended that the intake of CLA reach at least 3.4 g/day, which theoretically can reduce the percentage of fat in body and increase lean mass without side effects simultaneously [97].

## 14. Adverse Effects of CLA

Insulin resistance, increased lipid oxidation, and unsatisfactory serum lipid distribution including increased triglycerides, increased LDL-cholesterol, and decreased HDL content are all related to CLA supplementation [97].

## 15. *Momordica Charantia* L.

*Momordica charantia* L. (scientific name), the so-called bitter melon, belongs to the genus Momordica and Cucurbitaceae family. This plant is also known as balsam pear (English), bitter squash (English), bitter gourd (English), balsam apple (English), concombre africain and margose (French), balsambirne (German), balsamito (Spanish), peria (Malay), paria pare (Indonesian), and karalla (India). 

Bitter melon is an annual vine herb. It germinates best at high temperature, and it is a daily edible vegetable for Asian people. This plant grows in wild tropical and subtropical Africa, Asia, America, and the Caribbean. Moreover, it can also be cultivated [99].

Phytochemicals are rich in the fruits and leaves of bitter gourd. This plant is frequently applied in complementary and folk medicine to treat diabetes, hypertension, obesity, cancer, and bacterial as well as viral infections [100]. In Ayurvedic medicine, every single part of a plant such as seeds, roots, leaves, and especially immature fruits are used for medical applications. The juice is adapted for multiple treatment protocol in joint pain relief, fever, jaundice, liver diseases, as well as skin burns and rashes. Additionally, the entire plant can be used to treat diabetes. In Turkish folk medicine, the oil derived from the ripe fruit, soaked in olive oil, heated by sunlight, is mixed with honey to stabilize gastric ulcers. In African folk medicine, bitter gourd is mainly used for worm infections, inflammation (fruit, seed, and leaf juice), fever, menstruation (leaf), syphilis, rheumatism, and skin disease (root). In the Caribbean, it is administered in the form of leaf decoction or fruit juice to treat diabetes. The leaf decoction is also used to treat high blood pressure and worm infections [101].

As a natural antioxidant, bitter melon is a good source of phenolic compounds. Researchers have confirmed that bitter gourd is rich in alkaloids, steroidal glucosides, phenolics, lysophosphatidylcholines (LPC), CLNA isomers, and cucurbitane-type triterpenoids. The favorable effects of bitter gourd come from chemical components including cucurbitane-type triterpenoids, cucurbitane-type triterpene glycoside, phenolic acids, flavonoids, essential oils, fatty acids, amino acids, sterols, saponin constituents, and proteins. The pulp of this plant has a higher antioxidant activity than that of the seeds, which may be attributed to the different extents of phenolic acids and flavonoids [102]. Current studies have confirmed that bitter gourd has the therapeutic potential of lowering blood sugar, and lowering blood lipids, as well as anti-inflammatory, antioxidant, and anti-carcinogenic effects [103,104,105].

## 16. *Momordica Charantia* L. *var. Abbreviata Ser.*

*Momordica charantia* L. *var. abbreviata Ser.* (scientific name) is included in the family Cucurbitaceae. This natural plant is commonly named as wild bitter melon. *Momordica charantia* L. *var. abbreviata Ser.* is a wild form of *Momordica charantia* L. This plant is also known as shan ku gua (Mandarin), wild bitter melon (English), wild bitter gourd (English), kakorot (English), and balsampear (English). It is an annual vine. The size of its plant is smaller than that of bitter melon (about one-fifth) [74]. The color of the plant varies from green to dark green and has a strong bitter taste. In Europe and Asia, WBM can generally be used as a medicinal herb in folk medicine to treat a variety of diseases such as diabetes [106], alcoholic fatty liver [107], and to reduce blood pressure [100].

The bitter taste of bitter melon is derived from triterpene glycosides and cucurbitacin-like alkaloids. As a rule, immature bitter melon with darker green skin is more bitter, while bitter melon with lighter skin is less bitter. Furthermore, WBM has much higher saponins than bitter melon [102].

Pharmacologically, WBM has been addressed for its anti-inflammatory, antioxidant, anti-hyperglycemic, and anti-infectious properties [108]. The anti-inflammatory and antioxidant effect of WBM is better than that of bitter gourd [109]. LPS-stimulated RAW 264.7 macrophages showed a decrease in production of proinflammatory PGE2, iNOS, and COX2, along with attenuation of NFĸB activation after the administration of WBM. Moreover, WBM has been shown to scavenge free radicals [110] including 2,2-diphenyl-1-picrylhydrazyl (DPPH) and hydroxyl radicals [74]. 

### 16.1. α-ESA of WBM 

CLNAs are derived from the seed oils of WBM. As discussed, α-ESA, the isomer of CLNA is widely allocated among members of the family Cucurbitaceae, in particular WBM and bitter gourd. α-ESA is responsible for greater than 60%, and 30% of the total fatty acid in the seed oil is composed of bitter melon [111] and WBM [112]. It also accounts for around 19% of the total fatty acid composition of WBM ethyl acetate extracts. In dried and fresh WBM, the amount of α-ESA is 7.1 g/kg and 0.42 g/kg, respectively [112].

### 16.2. Inhibitory Effects of α-ESA on NFκB

CLA has been demonstrated to act as the ligand of PPAR-β [113]. As previously mentioned, it is clearly confirmed that α-ESA can be metabolized to CLA. α-ESA can thereby be used as a natural ligand for PPAR-β. The ligation of α-ESA and PPAR-β results in the retardation of IκB degradation and NF-κB activation [109]. PPAR-β has been shown to downregulate the secretion of transforming necrosis factor-α (TNF-α) in cardiomyocyte culture (via NF-κB inhibition) [47]. The PPAR-β agonist GW0742 can prevent IκB degradation in a mouse model of bleomycin-induced lung injury (via NF-κB inhibition) [48]. WBM has been shown to attenuate inflammatory responses in LPS-challenged RAW 264.7 macrophages by inhibiting NFκB activation [109].

### 16.3. CISD2-Elevating Effect of α-ESA in WBM

#### 16.3.1. In Vitro LPS-Challenged Neural Cells

LPS-challenged ALT astrocytes incubated with α-ESA demonstrated upregulated injury-attenuated CISD2, along with decreased GAFP and proinflammatory cytokines, when compared to LPS-incubated cells without treatment [10].

#### 16.3.2. In Vivo Mouse Model of Acute SCI 

WBM increased injury-attenuated expression levels of CISD2 along with reduced GFAP expression, as indicated in glial deactivation, and suppressed the PPAR-β/IκB/NF-κB signaling pathway [10].

## 17. Passage into the CNS of PUFAs and Conjugated Fatty Acids

PUFAs [79] and CLA isomers [114,115] have been shown to cross the blood–brain barrier (BBB). Evidence clearly demonstrates that PUFAs [116] and CLA isomers [117] penetrate into the cerebrospinal fluid (CSF). The results of research on humans indicate that PUFAs can be transferred from the bloodstream to BBB and CSF, thereby affecting the fatty acid concentration in the CNS [116]. As a result, the acquisition of PUFAs as well as PUFA-derived metabolites such as CLA and CLNA (via CLA conversion) by an enteral or systemic route will reach and cross the BBB, leading to beneficial effects in the CNS.

## 18. Polyphenols

Phenolic compounds are widely derived from nature. The key origins of phenolic compounds are from fruits, vegetables, and bark of woody vascular plants. According to chemical structure, phenolic compounds can be are divided into the following categories: phenolic acids, flavonoids, stilbenes, and lignans [118]. Phenolic compounds, flavonoids, and resveratrol from stilbenes are the main antioxidative compounds of plant foods [119].

Through donation of hydrogen atoms, phenolic compounds make free radicals form a stable resonance pattern, thereby inhibiting chain reactions of free radicals. Accordingly, it can chelate with metals, thereby slowing down the progress of oxidation [120]. When phenolic compounds inhibit the formation of ROS, they can inhibit inflammation, platelet aggregation, cell apoptosis, and enhance anti-inflammatory effects [121].

## 19. Curcumin as Polyphenolic Phytochemical

**Figure 3:**
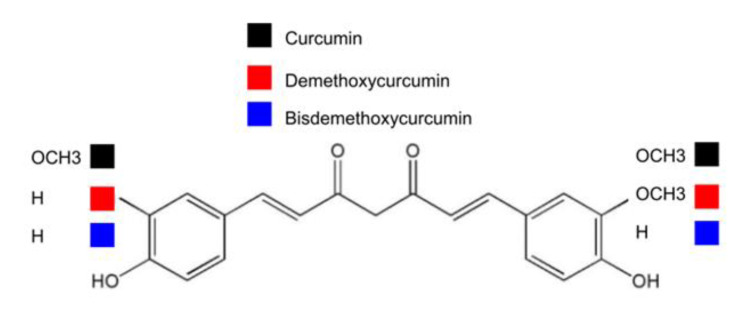
Chemical structure of curcuminoids: curcumin, demethoxycurcumin, and bisdemethoxycurcumin.

Curcumin [1,7-bis(4-hydroxy-3-methoxyphenyl)-1,6-heptadiene-3,5-dione] is a natural compound derived from turmeric (from the root and rhizome of the plant *Curcuma longa* L.). This flowering plant, curcuma longa, belongs to the Ziangiberaceae family, and is used as an alternative medicine for arthritis [122]. Turmeric is mainly composed of three curcuminoids including curcumin, demethoxycurcumin, and bisdemethoxycurcumin [123]. The three categories of curcuminoids are compared in structure. As indicated in Figure 3, two, one, and none of the phenyl methoxy groups can be detected in curcumin, demethoxycurcumin, and bisdemethoxycurcumin, respectively. With the phenolic O-H group, curcumin thereby belongs to the phenolic compound in structure. The antioxidant properties of curcumin can be achieved through the structure of hydroxyl groups and phenolic rings [123,124].

Curcuma longa have been widely used as seasonings in many ethnic cuisines in many countries, e.g., Bangladesh, India, and Pakistan. This compound has long been used as anti-inflammatory treatments in traditional Chinese and Ayurvedic medicines [122]. Curcumin, derived from Curcuma longa, is also popular in food circles as a dietary pigment and Indian spice. Pharmacologically, curcumin has been addressed for its anti-inflammatory, antioxidant, anti-carcinogenic, anti-infectious, hypocholesterolemic, and immunomodulatory activities [125,126]. Curcumin has been shown to regulate a variety of critical molecular targets, such as transcription factors, enzymes, cell cycle proteins, receptors, and cell surface adhesion molecules [127,128].

### 19.1. Toxic Dosage of Curcumin

When the content of curcumin-based essential oil complex exceeds 5000 mg/kg, no acute toxicity is shown in mice [129]. Curcumin has been shown to have no toxicity at doses exceeding 2 g/kg body weight in rats [130]. 

No side effects have been shown following the use of 8000 mg curcumin daily for three months [131]. In human clinical trials, the safe dose of curcumin has been addressed to be 10 grams per day [132]. Specifically, the acceptable daily intake (ADI) of curcumin is 0–3 mg/kg body weight (reports of the Joint United Nations and World Health Organization Expert Committee on Food Additives, JECFA; European Food Safety Authority, EFSA) [133].

### 19.2. Adverse Effects of Curcumin

Patients may experience nausea, diarrhea, increased serum alkaline phosphatase, and lactate dehydrogenase when ingesting over 450–3600 mg curcumin daily for 1–4 months. If one takes a single-dose of 500–12,000 mg (dose response study), symptoms and signs such as diarrhea, headache, skin rash, and yellowish stool can occur [133]. Further gastrointestinal discomfort, dyspnea, skin itching, and swelling may arise following high-dose curcumin intake [132].

### 19.3. Anti-Inflammation of Curcumin

Curcumin has been shown to decrease injury-induced neuroinflammation. Injury-triggered inflammatory mediators such as the pro-inflammatory transcription factor activator protein-1 (AP-1) and iNOS can both be attenuated after curcumin administration [134]. Injury-stimulated glial activation is indicated as expression of glial fibrillary acid protein (GFAP) [135] and production of injury-induced RANTES in astrocytes which is reduced in rats following SCI [136]. Lastly, curcumin impaired the activation of microglia and astrocytes and decreased the apoptosis induced by exposure to ozone (O3) in the hippocampus of rats [137].

### 19.4. Prevention of Mitochondrial Dysfunction of Curcumin

Ultraviolet irradiation-induced apoptosis, e.g., impaired mitochondrial membrane potential, cytochrome C release, and ROS production can be prevented after curcumin treatment [138]. Curcumin has been shown to scavenge ROS via electron donation from the phenolic hydroxyl group [139]. The antioxidant effects of curcumin involved the decrease in lipid peroxidation and scavenging of NO [140].

### 19.5. Inhibitory Effect of Curcumin on NFκB 

Curcumin has been well documented for antagonization of NFκB activation, along with inhibiting the aggregation of β-amyloid [141] and preventing the production of proinflammatory mediators, such as AP-1, TNFα, IL1β, and iNOS [134,142].

### 19.6. CISD2-Elevating Effect of Curcumin

#### 19.6.1. In Vivo Mouse Model of Aging

Curcumin-managed mice demonstrated a remarkable elevation in CISD2 protein expression in the spinal cord, in contrast to their unmanaged counterparts [13].

#### 19.6.2. In Vitro Neural Cell Model of Aging 

Curcumin-treated extreme aging astrocytes (35 *DIV*) demonstrated CISD2-elevating effect and reduced extent of neuroinflammation, as indicated in an attenuation of iNOS and RANTES expression levels, when compared to cells untreated with curcumin [13].

#### 19.6.3. In Vivo Mouse Model of Acute SCI 

Curcumin treatment can augment injury-attenuated CISD2 expression as well as CISD2-positive cells in mice following SCIs [12].

#### 19.6.4. In Vitro LPS-Challenged Neural Cells

LPS-challenged astrocytes with curcumin treatment demonstrated a significant increase in CISD2 expression when compared to LPS-challenged astrocytes untreated with curcumin [12].

## 20. Passage into the CNS of Curcumin 

The following evidence proves that curcumin plays a protective role in CNS. (1) Curcumin passes easily into the BBB [141]. (2) The ability to increase the permeability of the BBB during brain ischemia [143]. (3) The ability to enhance the integrity of the blood–spinal cord barrier after SCI [144]. (4) The percolation of curcumin into the CSF [145]. CSF circulates in the subarachnoid space around the CNS, so curcumin is delivered with ease to the brain and spinal cord.

## 21. CNS Pathology-CISD2-NFκB Axis and Perspectives

In the CNS, glial cells can be abnormally activated during aging, neurodegenerative disease, and neurotrauma. Glia-mediated neuroinflammation and mitochondrial dysfunction along with CISD2 decline and NFκB activation are implicated in CNS injuries and diseases. CISD2, as a NEET and iron-sulfur protein with a [2Fe-2S] cluster, has been shown to modulate various important proteins, such as Bcl2, PPAR-β, and NFκB. CISD2 can regulate critical physiological functions, such as apoptosis or mitochondrial Fe/ROS/Fe-S homeostasis. The unique characteristic of this protective protein is highlighted throughout this article, acting as an NFκB antagonist. Previous in vitro data showed that siCISD2-transfected neural cells (untreated or treated with LPS) commonly exhibited inflammatory responses, pronounced mitochondrial dysfunction including lower DeltaPsi (m), higher ROS levels, more extensive apoptosis, and a reduction in cell survival. Since NFκB mediates pro-inflammatory cascades and mitochondrial function, injury-driven pathological decline of CISD2 undoubtedly weakens the inhibitory effect on NFκB activation, causing a wide range of harmful effects.

Under circumstances of CNS injuries and diseases, how to elevate insult-attenuated CISD2 expression holds promises for treatment. Phytochemicals from natural compounds beneficial to CISD2-elevation can be considered and applied to manage CNS injuries and diseases. Natural phytochemicals, α-ESA (CLNA isomer from WBM) and curcumin, both are able to enter the CNS through the BBB, which is conducive to this axis of CNS pathology–CISD2-NFκB (Figure 1). The above two phytochemicals, exerting neuromodulatory effects on CISD2 elevation, can be considered to be applied in CNS injuries and diseases.
